# Extending lifespan: Wear and non-wear component analysis in end-of-life products

**DOI:** 10.1016/j.mex.2025.103423

**Published:** 2025-06-07

**Authors:** Waqas Ahmed, Jenny Bäckstrand, Vanajah Siva, Niklas Sarius, Hans-Åke Sundberg

**Affiliations:** a Husqvarna Group, Huskvarna, Sweden; b Department of Supply Chain and Operations Management, School of Engineering, Jönköping University, Jönköping, Sweden; c SealEco International, Värnamo, Sweden

**Keywords:** Circular economy, Wear and tear analysis, Sustainable consumption, Component condition, R-strategies, 360° visual diagnostic tool on Product- component level

## Abstract

Products often end up in landfills after serving their purpose, e.g. after their end-of-life. While some parts can be/sometimes are salvaged for valuable materials like rare earth elements, the majority are discarded—a common but wasteful practice. The concept of circular economy promotes recycling or recovering to maximize resource efficiency and to avoid landfill from end-of-life products, though often sub-optimally. Our method introduces a simple, yet novel, concept grounded in circular economy principles to extend the lifespan of end-of-life products’ components. The product is disassembled, and components are grouped based on their susceptibility to wear and tear. Using a 360° visual diagnostic tool, individual components are then categorized as either healthy with reduced remaining-useful-life or having reached end-of-life. Appropriate R-strategies such as reuse, repurpose, refurbish, remanufacture, recycle, and recover are subsequently applied, extending the lifespan of the component. The proof-of-concept for the 360° visual diagnostic tool is validated using a product from a Swedish outdoor power product manufacturer.

• Focus on end-of-life products not originally designed with circular economy principles.
• Expands R-strategies from recycle and recover to reuse, repurpose, refurbish, remanufacture, recycle and recover for end-of-life products.
• Provides practitioners with a decision-making tool to estimate component-level circularity and identify end-of-life strategies.

## Graphical abstract

**Figure:**
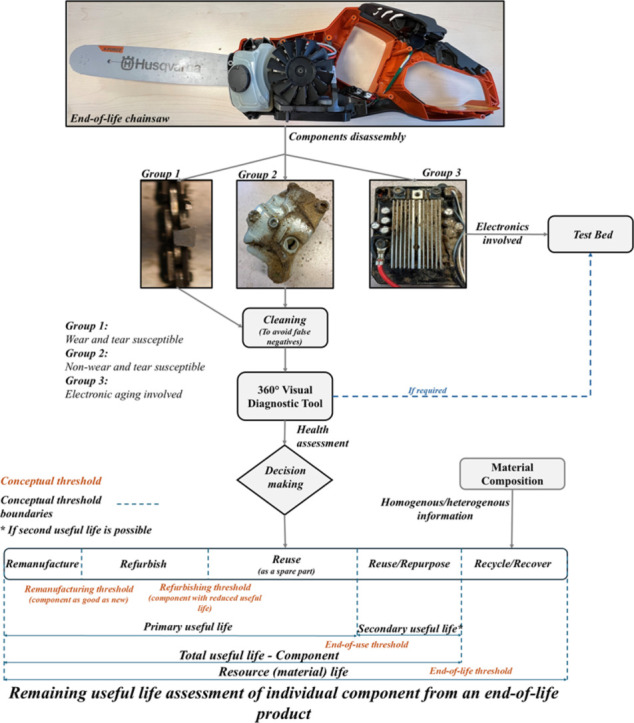

Specifications table

**Table:** 

Subject area:	Engineering
More specific subject area:	End of life management for extending circular economy practices
Name of your method:	360° visual diagnostic tool on Product- component level
Name and reference of original method:	W. Ahmed, V. Siva, J. Bäckstrand, N. Sarius, H. Å. Sundberg, Circular economy: Extending end-of-life strategies, Sustainable Production and Consumption 51 (2024) 67-78. https://doi.org/10.1016/j.spc.2024.09.003
Resource availability:	Equipment: 540i XP® (battery and charger included) - Husqvarna (Available at: https://www.husqvarna.com/us/chainsaws/540i-xp-battery-and-charger-included/?article=967864003) [1].

## Background

The current global push towards sustainability has highlighted the limitations of traditional linear economic models, which typically involve the production, use, and disposal of products. The circular economy (CE) concept, which emphasizes the reuse, repair, refurbishing, and recycling of products and materials, offers a promising alternative [2,3].

Products that have reached their end-of-life (EoL), particularly those not originally designed with CE principles in mind, often end up in landfills. This not only contributes to environmental pollution but also represents a significant loss of valuable materials [4]. Research has shown that a large proportion of these products are discarded despite containing components that could be repurposed or recycled [5]. Traditional EoL strategies often involve straightforward recycling or disposal, which fails to maximize resource efficiency. Adopting more sophisticated approaches to EoL products can significantly enhance their lifespan value [6,7]. However, existing methods lack the precision needed to distinguish reusable and non-reusable components efficiently at a micro level.

Therefore, in this article, we build upon the research by Ahmed et al. [6] and further extend their work by introducing additional use-phase R-strategies to EoL products at the component level. This approach aims to optimize the existing EoL strategies; recycle and recover, by incorporating remanufacture, refurbish, reuse, repurpose, recycle, and recover strategies.

**Fig 1:**
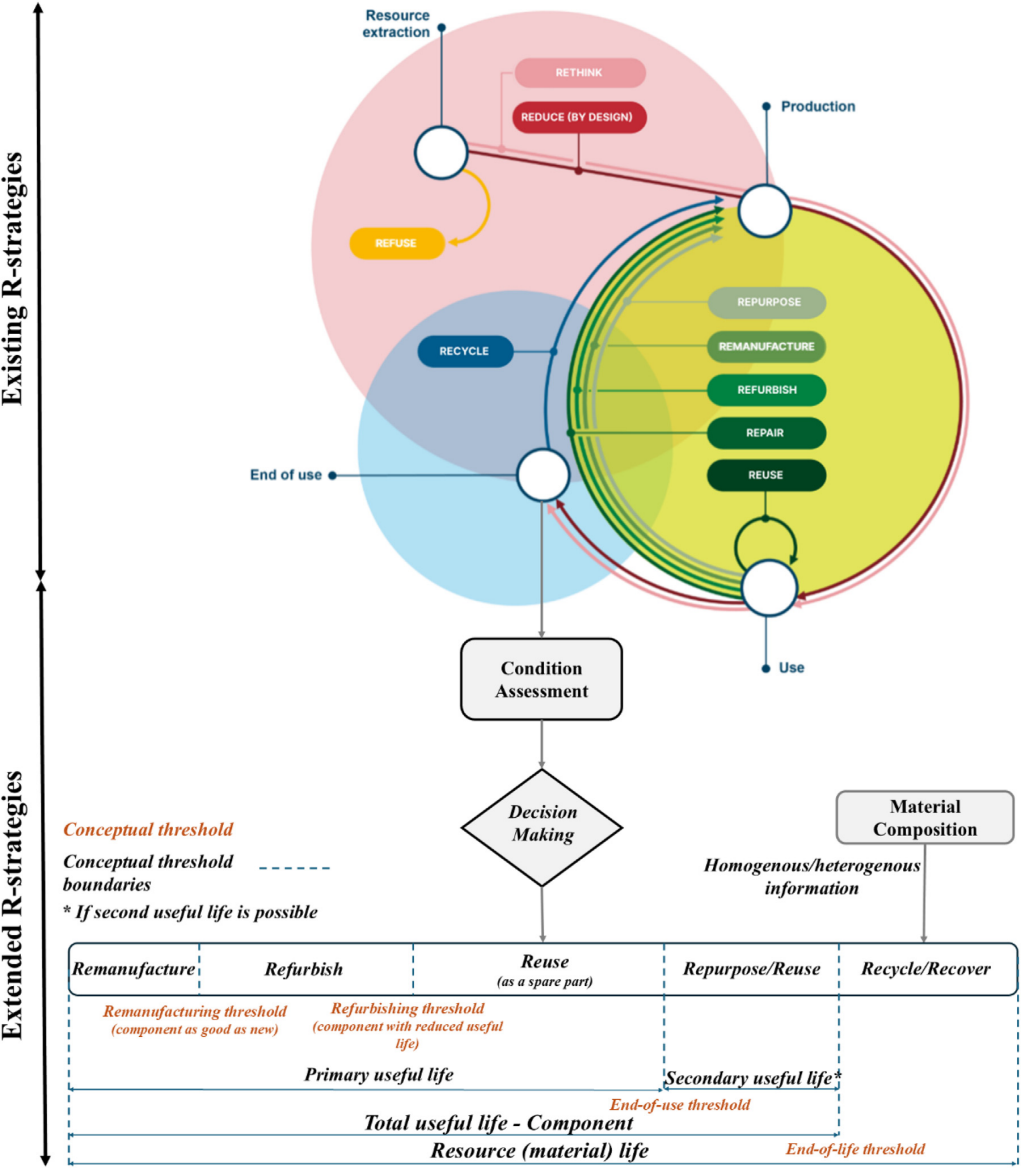
Extending R-strategies at EoL – Inspired by Deutsches Institut für Normung [8].

Fig. 1 illustrates the existing R-strategies for EoL products, along with the proposed extended use-phase R-strategies at the component level. Relevant R-strategies are recommended based on the healthy component's useful life. If a component has a reduced useful life, it can be repurposed if there is a secondary application (such as batteries); otherwise, it is reused as a spare part (e.g., screws, nuts) until it reaches the end of its useful threshold. At this point, either recycle or recover strategies are employed, based on the component's composition (detailed later).

For this purpose, a simple yet novel tool to evaluate the EoL product components is proposed. The tool, the 360° visual diagnostic tool, is evaluated on an EoL product from a large outdoor power products manufacturer, a battery chainsaw (BCS). The proposed 360° visual diagnostic tool is designed to consider the individual component complexity, such as wear and tear susceptibility and electronics, and offer relevant analysis. It can either propose a detailed test bed for health analysis or subject the components to a 360° visual analysis against the representation of a healthy (before use) component to detect anomalies. In the case of an unhealthy component (having reached its EoL), the tool provides composition complexity (homogeneous or heterogeneous) to extend the relevant R-strategy, i.e., recycle or recover.

This approach facilitates the transition towards a CE for existing products while considering their limitations and complexities. In broader terms, it will extend the lifespan of components with reduced remaining useful life, significantly reducing landfill use and the environmental impact associated with product disposal.

## Method details

**Fig 2:**
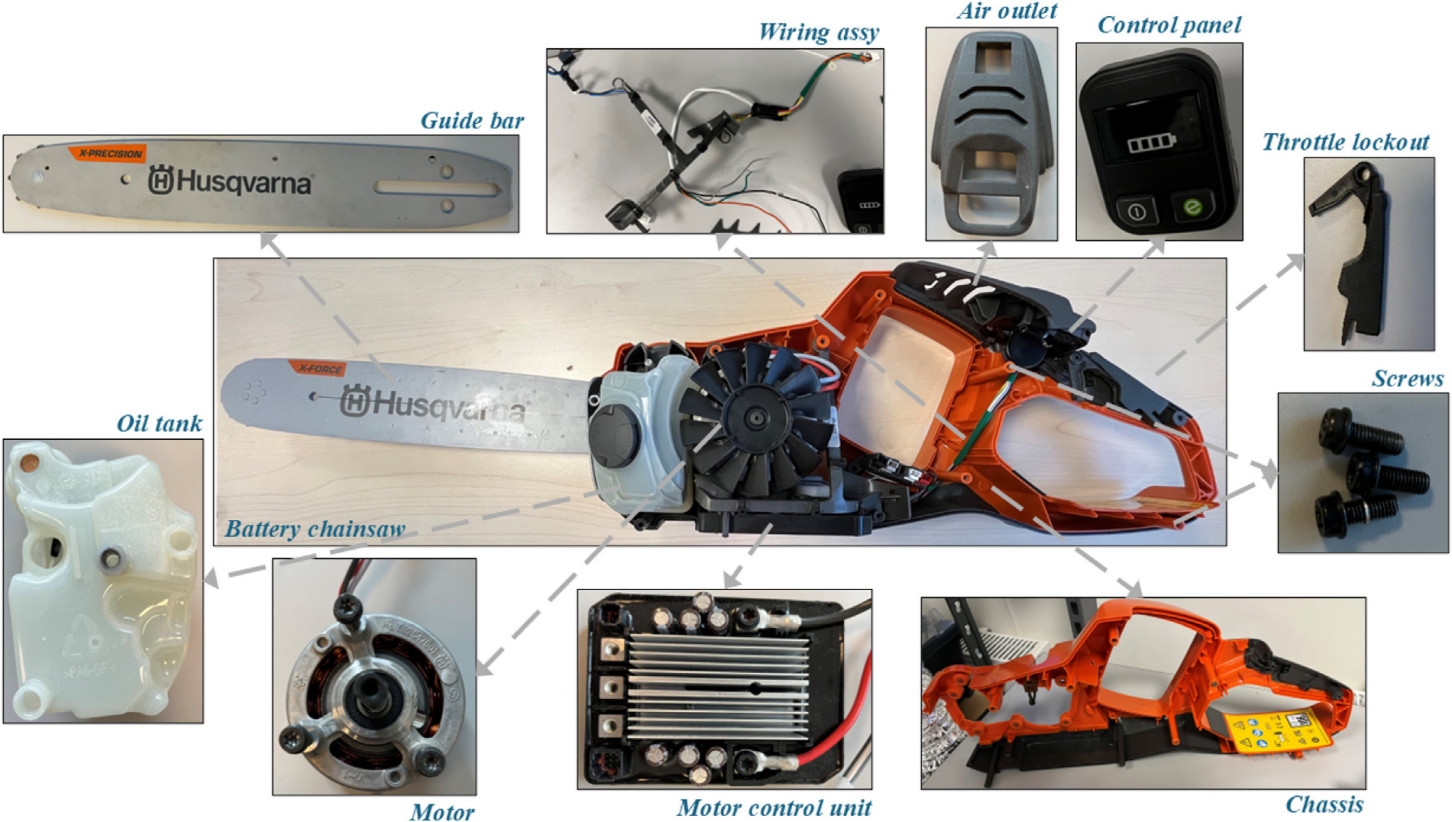
BCS components, excluding battery.

A BCS consists of multiple components that work together to ensure high efficiency and cutting performance. Each component is made from different materials, such as plastic, copper, and magnesium, and varies in complexity, being either homogeneous or heterogeneous. This design takes into account multiple factors, including susceptibility to wear and tear, while ensuring the BCS is lightweight and easy to handle [9]. Each component serves a specific purpose: the motor drives the chains to make cuts, the motor control unit powers the motor to maintain the necessary RPMs according to the log hardness, and the wiring assembly connects the electrical components, including the battery, motor control unit, and motor. The oil tank lubricates the chains and guide bar to reduce unnecessary power loss due to friction. Additionally, the chassis houses all the components. Fig. 2 illustrates some of the components of a BCS for reference.

Once a BCS reaches its EoL, it is collected. Rather than categorizing the entire BCS as EoL, a diagnostic method is introduced at the product component level. The BCS is then disassembled into its individual components to assess their condition. This approach extends the lifespan of healthy components with reduced remaining useful life through remanufacturing, refurbishing, reusing, or repurposing, rather than treating these healthy components as if they have reached the EoL stage.

The CE concept typically recommends either recycling or recovering strategies after product EoL [10], which is suboptimal and reduces resource efficiency. Additionally, individual components are categorized into three groups based on their function and composition:

1. Wear-and-tear susceptible components (Group 1)
2. Non-wear susceptible components (Group 2)
3. Electronics aging components (Group 3)

**Fig 3:**
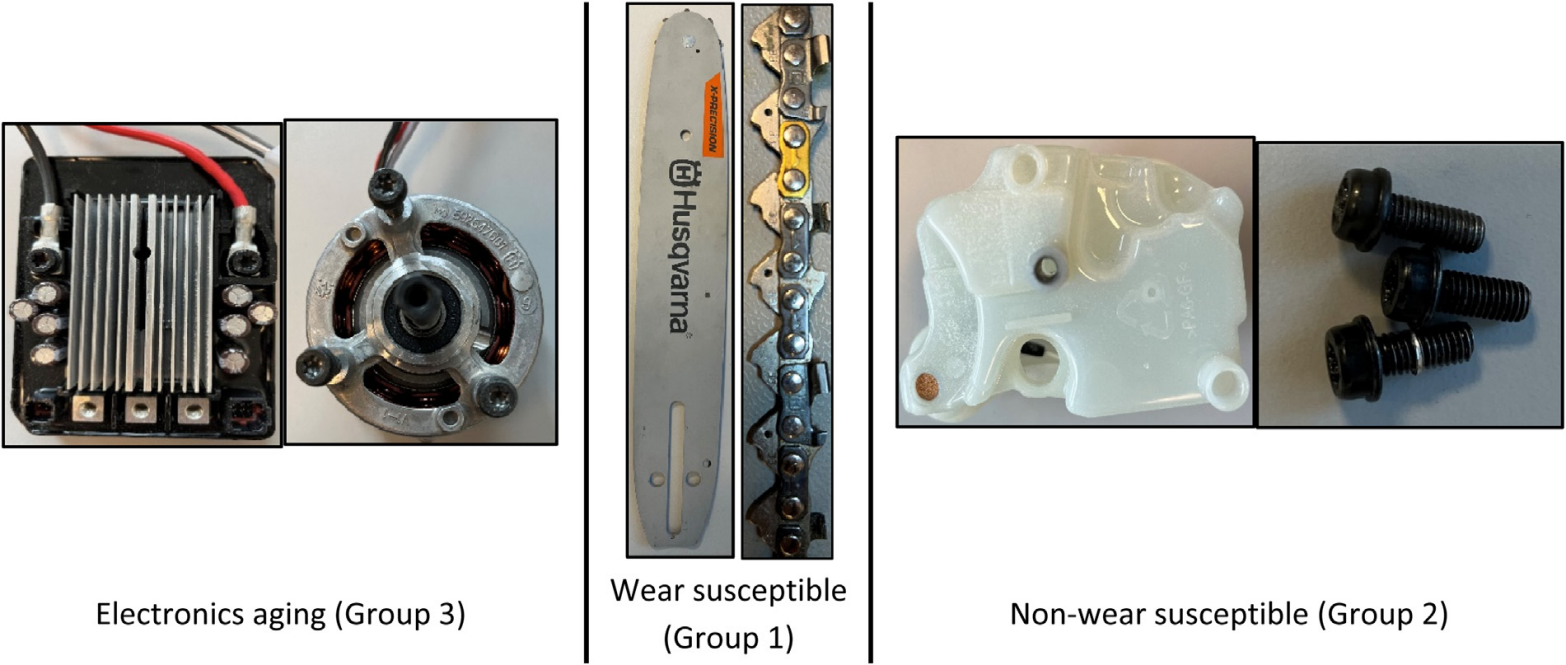
BCS components – Based on function and composition.

This categorization enables a more strategic approach to lifespan extension, ensuring that each group receives targeted solutions that address its unique characteristics. Fig. 3 provides a visual representation of these categories for better understanding.

Group 1 and 2 components constitute approximately 70% of the BCS components [6]. A 360° visual diagnostic tool can be applied to these components based on their visually detectable condition. To ensure accurate surface condition analysis, each component is first cleaned, minimizing the risk of false negatives. After cleaning, the component is compared against a stored 360° representation of a healthy (before use) component in a database, allowing for precise deterioration assessment.

The utility of each component is determined through a structured comparison between the used component and its 360° representation of a healthy (before use) component stored in a database. In this study, this comparison is conducted manually by product expert (BCS expert) as part of a proof-of-concept. The decision-making process includes the following steps:

• Each component is cleaned to remove surface contaminants that may interfere with visual analysis.
• The component is visually compared from all angles to a 360° representation of a healthy (unused) component stored in a database. For example, a used oil tank is compared to a 360° representation of a healthy (unused) oil tank. The 360° representation of a healthy (unused) component reflects the original geometry and surface condition. The comparison focuses on identifying deviations such as wear depth, edge rounding, leakage, deformation, cracks, and surface roughness, depending on the component’s function and criticality.
• A component is considered reusable if the deviation remains within a component-specific tolerance range, defined by experts. For instance:
  ○ Oil tank: up to 15% deviation
  ○ Chains: up to 60% deviation
  ○ Screws: typically below 10% deviation

(These thresholds were set for this study).

• Based on the deviation, the component is assigned to an appropriate R-strategy. For example, in the case of oil tanks:
  ○ < 2% deviation: Remanufacture potential
  ○ 2–5% deviation: Refurbish potential
  ○ 5–15% deviation: Reuse as spare part or repurpose for a less critical application (if repurpose is possible)
  ○ >15%: Recycle or recover, depending on material composition

This mapping is not fixed but adapted based on the component’s role and safety requirements. For instance, a 10% deviation may be acceptable for a non-critical plastic housing but not for a high-stress mechanical part.

• When the 360° visual diagnostic tool cannot conclusively assess a component typically due to ambiguous surface features, edge anomalies, or for quality assurance, detail analysis is conducted such as microscopic study. The procedure is currently manual and performed by expert using standard optical microscopy. The required time varies depending on the component's complexity and the capabilities of the handling person or system. In this case:
  ○ Screws: approximately 5-10 minutes
  ○ Oil tank: approximately 10–12 minutes
  ○ Chains: approximately 15–20 minutes

**Fig 4:**
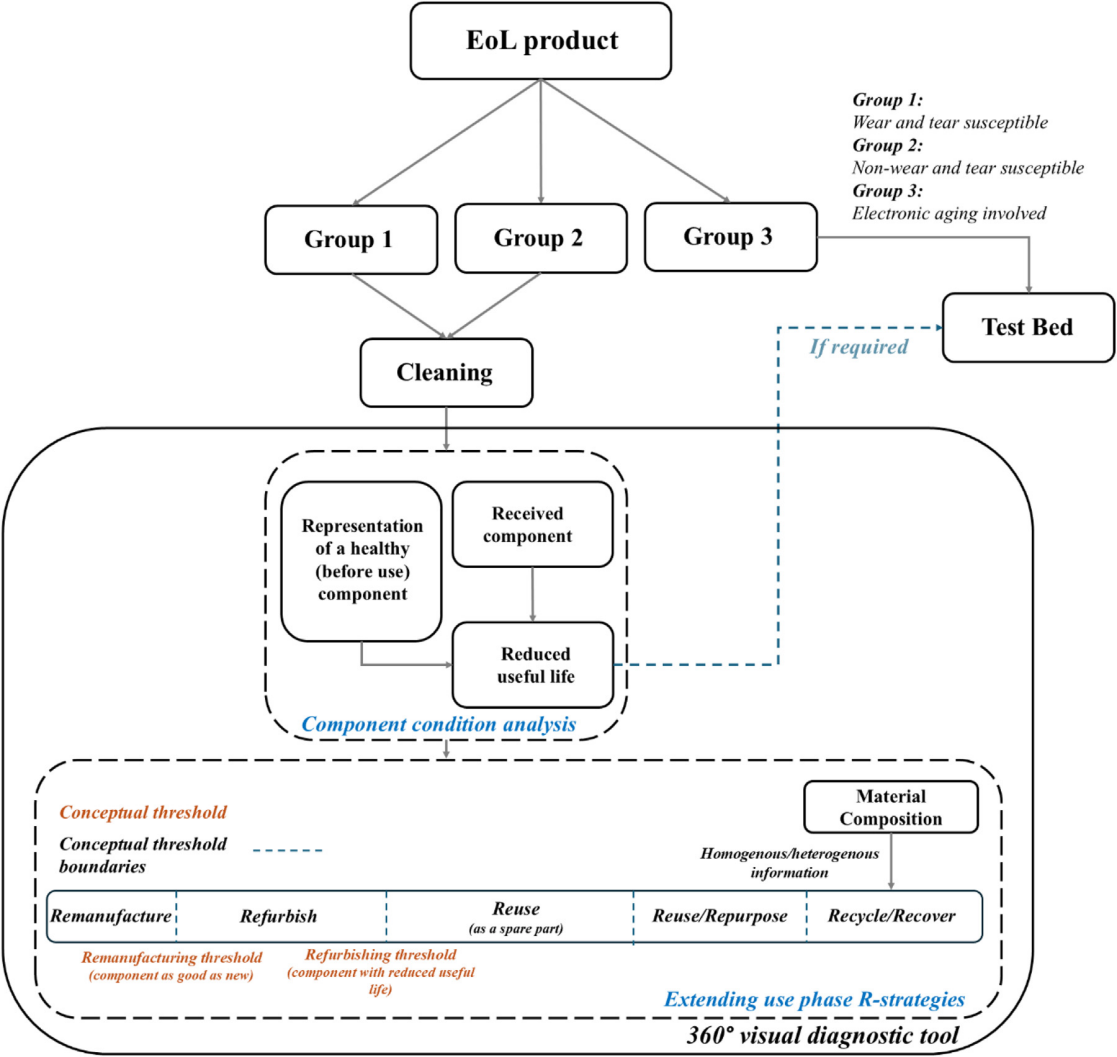
360° Visual diagnostic tool – Working framework.

The 360° visual diagnostic tool does not use a coincidence matching algorithm or automated similarity scoring. Instead, the comparison is conducted manually by expert (as part of a proof-of-concept). The decision-making process relies on expert judgment supported by visual inspection for this study. Fig. 4 illustrates the framework of the 360° visual diagnostic tool.

The group 3 components (electronic aging) are prone to natural and accelerated aging, which causes performance decline over time [11]. While the 360° visual diagnostic tool is useful for assessing surface condition, it is not suitable for determining whether an electronic component is functional or defective. Due to their electronic nature, a test bed is required to evaluate performance accurately. This ensures that components are tested under operational conditions to determine their actual functionality.

The decision to recycle or recover an unhealthy component primarily depends on its composition complexity, whether it is homogeneous or heterogeneous, due to technological limitations [12]. Components with homogeneous compositions are typically easier to process for recycling, while heterogeneous components may require advanced recovery techniques to maximize material reclamation. As for healthy components, however, extending the repurpose, reuse, refurbishment, or remanufacturing options involves multiple considerations. One instance is the existence of a secondary useful life or application for the component (such as screws, oil tank, motor, battery), and the reduced remaining useful life of each individual component (refer to Fig. 4), are crucial factors [6].

**Fig 5:**
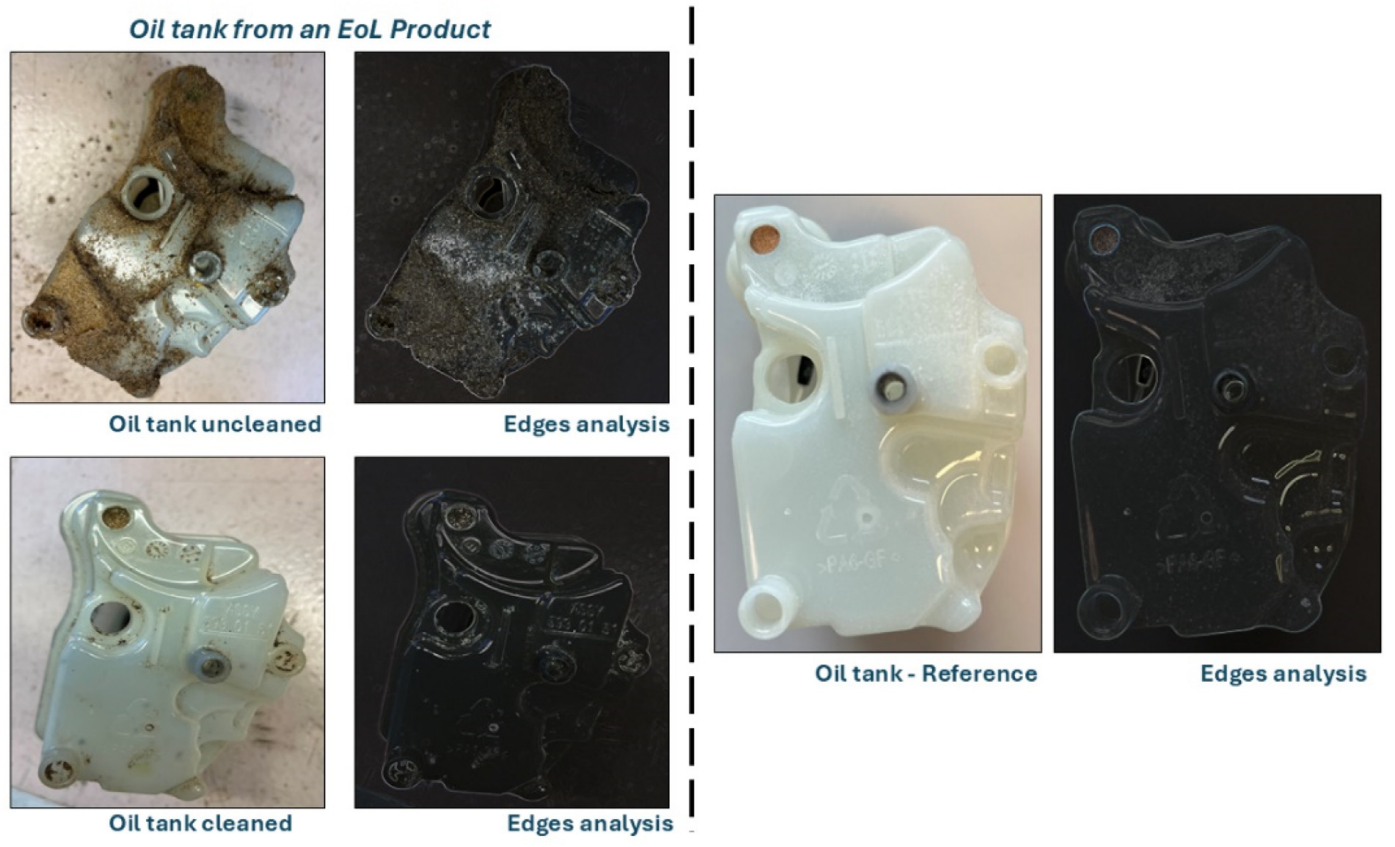
Oil tank analysis as an example – 360° visual diagnostic tool top view.

Fig. 5 illustrates the impact of EoL component cleaning on the performance of the 360° visual diagnostic tool when compared to the representation of a healthy (before use) component stored in a database, which may result in false negatives. Edge analysis is utilized for enhanced representation, offering valuable insights into wear patterns, material loss, surface roughness, and failure mechanisms. By analyzing these factors, edge analysis aids in predicting component lifespan and contributes to improved maintenance strategies and design optimization [13]. However, if any potential issues are detected in a component, it undergoes a more detailed testing setup to precisely identify and address the problems [6]. For instance, if the 360° visual diagnostic tool identifies an abnormal wear pattern on the edge of a component, the component undergoes further analysis (refer to Fig. 4) to assess its condition in greater detail. This additional examination ensures quality assurance by verifying performance and reliability before determining its next course of action.

## Method validation

**Fig 6:**
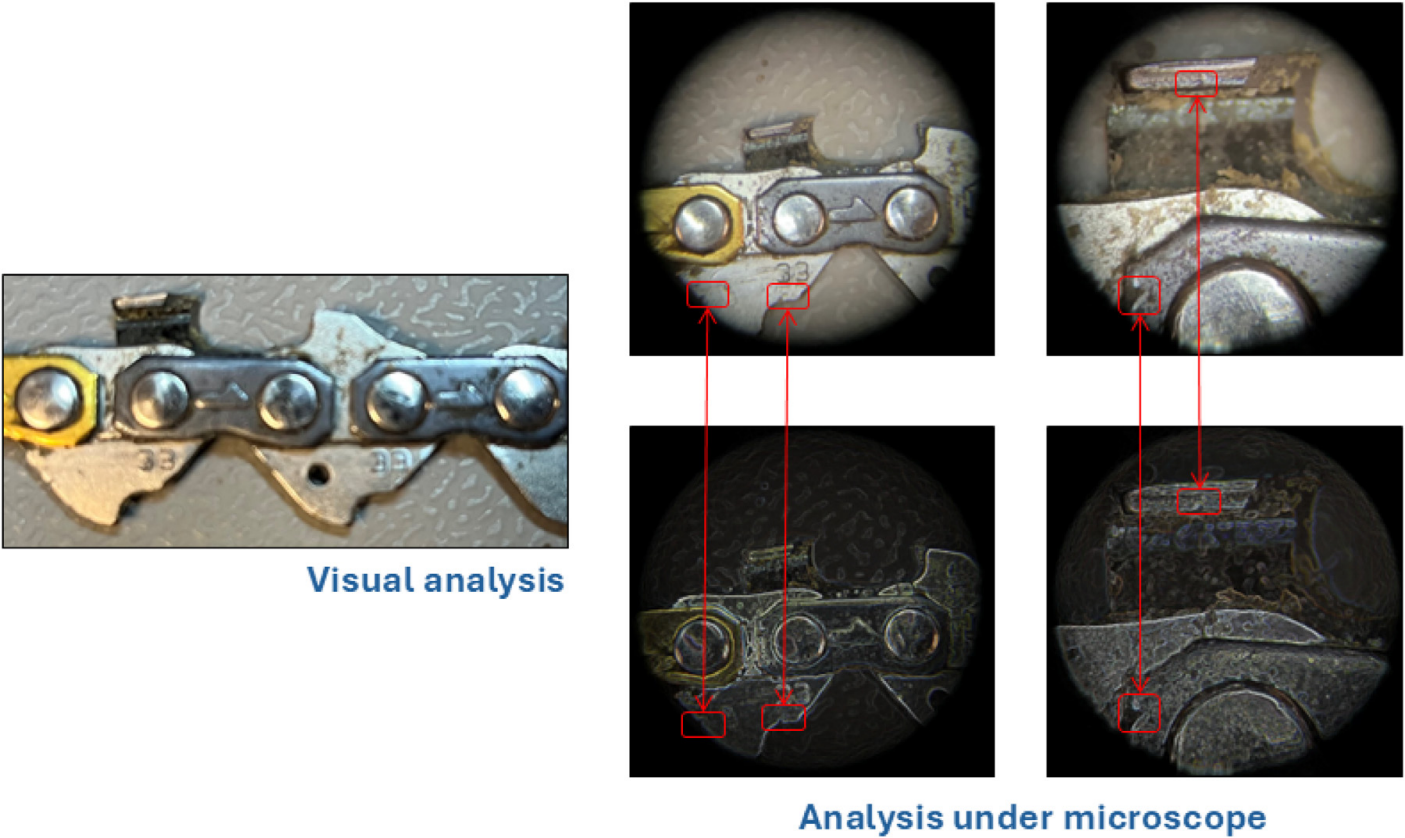
Wear and tear pattern on chains.

The application of the 360° visual diagnostic tool is validated using two simple components (with multiple samples) from Group 1 (wear) and Group 2 (non-wear): the chain and the screw. These components were selected due to their simplicity and homogeneous nature. Fig. 6 shows the wear and tear of a chain from Group 1 components, which, according to the 360° visual diagnostic tool, appears to be perfectly suitable for further logging purposes considering their top plate, rivets, chain link, etc. However, the length of the new chain's top plate is approximately 9mm when compared to a representation of a healthy (before use) component, stored in a database, indicating a reduced remaining useful life of the chain as shown in Fig. 6. Since chains are susceptible to direct wear and tear, a detailed analysis reveals some wear and tear patterns (indicated in red boxes, refer to Fig. 6; microscopic analysis is conducted as supporting evidence; however, it is not a component of the 360° visual diagnostic tool. Instead, it serves as an additional verification method, providing deeper insights into surface characteristics and material integrity beyond what is visually detectable). These marks were anticipated during their utilization. Nevertheless, breakage of chain links, which can pose a safety issue, is not observed. Therefore, the chains are still deemed perfectly suitable for logging purposes, as also indicated by field experts.

**Fig 7:**
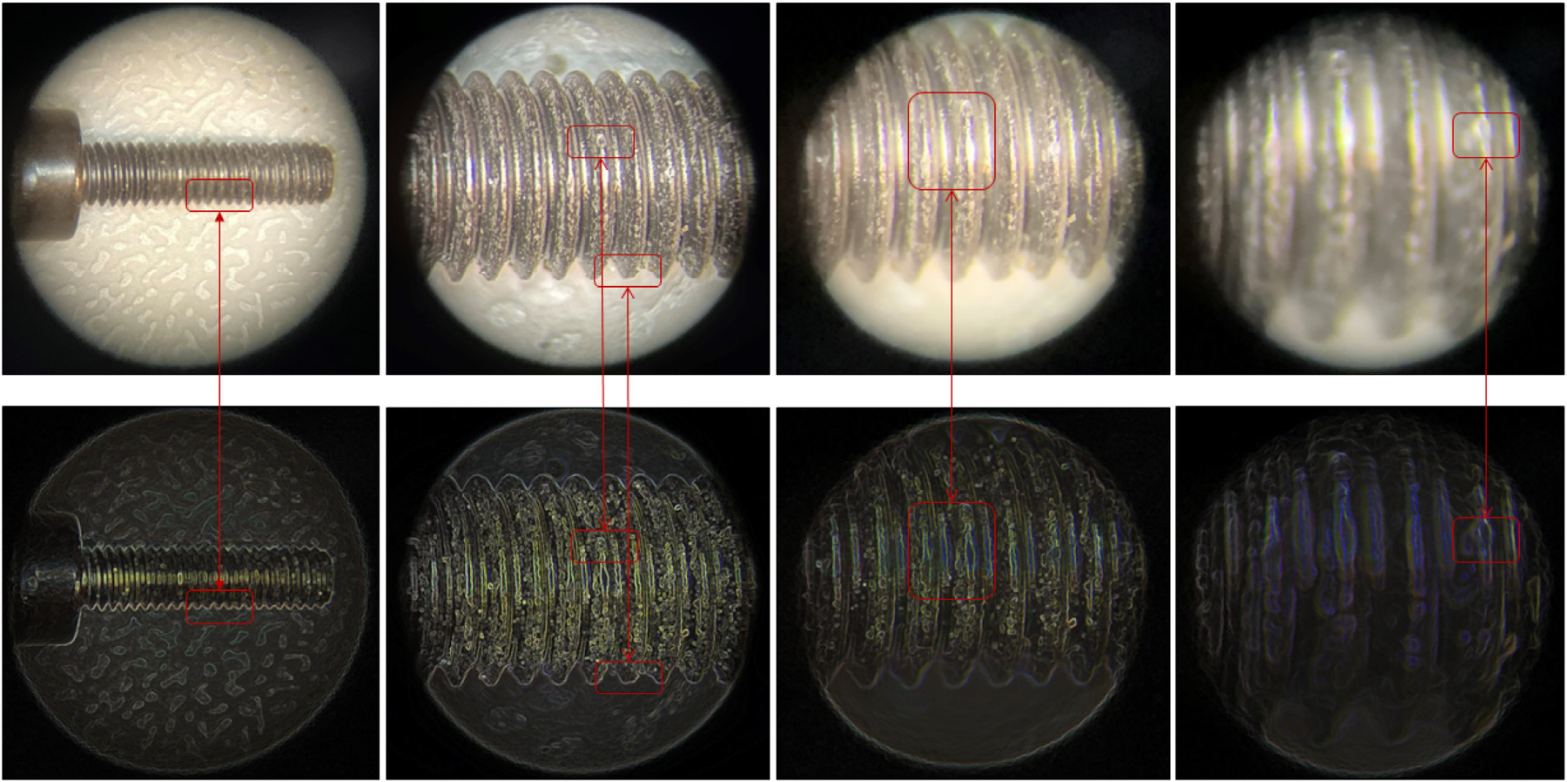
A screw reduced remaining useful life due to wear and tear.

In contrast, a Group 2 component, a screw, is not directly susceptible to wear and tear and can be reused almost indefinitely, as indicated by the 360° visual diagnostic tool when compared against the representation of a healthy (before use) component, stored in a database. However, a microscopic study reveals minute signs of wear and tear due to repeated tightening or could have been used as fastening for a component that vibrates and thus it might be worn, indicating a reduced remaining useful life. These minor imperfections may reduce the screw’s functionality over time, leading to un-tightened or slippage issues if subjected to similar conditions. These issues are identifiable through the 360° visual diagnostic tool, and such screws will be deemed unfit for further use once the wear pattern or damage reaches the tolerance values i.e. slippage. Fig. 7 provides a detailed analysis of these wear patterns, highlighted in red boxes through edge analysis.

## Limitations

Health-based categorization of EoL products at the component level extends and further optimizes CE EoL approaches, increasing resource efficiency. The use of a 360° visual diagnostic tool on outdoor power products, which are not designed according to CE principles, provides realistic applications in optimizing EoL approaches, also for other products where the level of wear and tear will differ between components. Additionally, an useful life analysis complements this approach, helping to narrow down the relevant R-strategies such as reuse, repurpose, refurbishing and remanufacturing, providing additional benefits.

However, there are certain limitations to this approach. Firstly, the analysis was conducted manually due to the absence of necessary infrastructure for collecting, disassembling, and analyzing components from EoL products. While the results were validated by field experts, the manual identification of individual components, particularly those in Group 1 and Group 2, is neither straightforward nor efficient. This process is time-consuming and labor-intensive (detailed previously), which restricts the early adaptability of the 360° visual diagnostic tool. Secondly, existing products are not typically designed to be easily disassembled. This limits the application of the 360° visual diagnostic tool, as many components are difficult to access and evaluate. Despite the benefits, the 360° visual diagnostic tool may face challenges in accurately assessing the health of complex or damaged components, leading to misclassification and suboptimal resource recovery strategies. Although this approach is environmentally sustainable, it is not yet economically viable due to the high capital investment required and the need for a robust reverse logistics network to efficiently collect EoL products. However, the ultimate goal is to achieve economic sustainability in accordance with EU regulations, ensuring both environmental and financial feasibility in the long term.

## CRediT authorship contribution statement

**Waqas Ahmed:** Conceptualization, Investigation, Formal analysis, Methodology, Visualization, Validation, Writing – original draft, Funding acquisition. **Jenny Bäckstrand:** Writing – review & editing, Supervision. **Vanajah Siva:** Writing – review & editing, Supervision. **Niklas Sarius:** Supervision, Validation. **Hans-Åke Sundberg:** Supervision, Validation.

## Declaration of competing interest

The authors declare that they have no known competing financial interests or personal relationships that could have appeared to influence the work reported in this paper.

## Data Availability

Data will be made available on request.
